# Malaria Control: Tortoises and Hares

**DOI:** 10.4269/ajtmh.15-0173

**Published:** 2015-05-06

**Authors:** Steven R. Meshnick

**Affiliations:** Department of Epidemiology, University of North Carolina Gillings School of Global Public Health, Chapel Hill, North Carolina

How long will it take to effectively control and then eliminate malaria in sub-Saharan Africa*?* Is it a sprint to the finish line or a crawl? Will the tortoise or hare win the malaria race?

As described in this issue, Kamya and others measured temporal trends in malaria over a 2 year period in three Ugandan sites with different transmission intensities.^1^ One hundred households were randomly selected from each site. Entomological inoculation rates (EIRs) were assessed monthly. All children aged 6 months to 10 years in each household were enrolled. Malaria was assessed by both active and passive surveillance. The three sites—Walukuba, Kihihi, and Nagongera—had a wide range of EIRs: 2.8, 13, and 310 infectious bites per year, respectively. Long-lasting insecticide impregnated net (LLIN) ownership was high at all three sites: 57.5%, 51%, and 78.5%, respectively. Reported LLIN utilization was very high, with over 99% of study participants reporting that they slept under bed nets the night before each visit (every 3 months) by the study team. Community coverage with artemisinin-based combination therapy for children with recent fever was also very high (75–82%). Thus, all three sites had very effective malaria control programs.

The results of the study were surprising in several ways. First, malaria incidence was remarkably high, ranging from 0.43 to 2.81 episodes per study participant per year. How could so many children get malaria if their reported bed net utilization was 99%? One possible explanation is recall bias. Study participants may have said they slept under a net because they thought it was the answer the interviewers wanted. Insecticide resistance (biochemical or behavioral) is another possibility. A third and likely possibility is that study participants benefitted from the nets, but that community transmission levels were so high that they nonetheless experienced malaria frequently.

Over 2 years, malaria incidence among participants fell in the low-transmission site (Walukuba). This is good news; malaria control efforts appeared to have had substantial positive effects when the initial transmission level was low. This observation is consistent with those in other low-transmission sites or island areas with relatively little imported malaria.

Regardless of malaria incidence, in the context of a cohort trial with rapid access to appropriate treatment, outcomes were excellent, with severe malaria seen in only 8 of 2,582 episodes and no malaria deaths. Thus, even before we come close to eliminating malaria, we can greatly curtail malaria deaths by providing rapid access to appropriate care.

The second surprise was that malaria incidence remained stable or increased at Kihihi and Nagongera, the two higher transmission sites. This occurred despite the fact that some mathematical models predicted that incidence should have decreased. Most of the world's falciparum malaria occurs in high-transmission areas akin to Kihihi and Nagongera. Does this mean that bed nets and case management don't affect malaria incidence when the transmission levels are high?

Malaria incidence and prevalence did not fall in Nagongera, the site with very high transmission intensity, despite the fact that 78.5% of households reported bed net ownership in 2012. This high level of bed net ownership, however, could be misleading. The high ownership level was probably the result of a mass bed net distribution campaign in 2011. But actual utilization could have been much lower. Conceivably, community members could have gradually stopped using the nets between the time they received them in 2011 and the time the study ended in 2013. Long-term follow-up adherence studies of mass bed net distribution campaigns are needed.

But the most likely reason for malaria's persistence is that control measures require more than 2 years to have an impact. In 1947, the incidence of malaria in Thailand was 286/1,000,^2^ higher than most of modern Africa. Now, malaria is relatively rare in Thailand, except in border areas. It took a concerted effort lasting more than 60 years for malaria control in Thailand to succeed. In other countries, malaria control programs were often thwarted by their own successes. Control programs had their budgets slashed just as they were succeeding in reducing malaria prevalence. The cuts frequently led to resurgent disease.^3^

There is a second reason why malaria control and elimination requires long-term investment. Many malaria control programs in sub-Saharan Africa are fragmented, district by district, province by province, or country by country. What happens when there is success in one district but all of its neighbors are still highly endemic? Diligence will be needed for years after successful malaria elimination programs to prevent reintroduction.

Unfortunately, funding for malaria is political and politicians have short attention spans. Politicians suffer from what the behavioral economists call “ego depletion” or being too busy responding to immediate impulses and stresses to make the right long-term decisions.^4^ Pitching a 10- or 20-year program to a politician is like asking a toddler to save his or her Halloween candy for later.

Hares may be better at attracting donor funds than tortoises, but, by raising expectations, they may ultimately cause disappointment and donor fatigue. As we learned from the Malaria Eradication Program of the 1950s, even reasonable donors eventually lose confidence when little long-term progress is made.

So the real take-home message of Kamya and ohers^1^ may be that malaria control in Africa requires sustained and consistent efforts over much more than 2 years. Hares beware.
